# A Systematic Evaluation of the Impact of STRICTA and CONSORT Recommendations on Quality of Reporting for Acupuncture Trials

**DOI:** 10.1371/journal.pone.0001577

**Published:** 2008-02-13

**Authors:** Stephanie L. Prady, Stewart J. Richmond, Veronica M. Morton, Hugh MacPherson

**Affiliations:** Department of Health Sciences, The University of York, York, United Kingdom; Canadian Agency for Drugs and Technologies in Health, Canada

## Abstract

**Background:**

We investigated whether there had been an improvement in quality of reporting for randomised controlled trials of acupuncture since the publication of the STRICTA and CONSORT statements. We conducted a before-and-after study, comparing ratings for quality of reporting following the publication of both STRICTA and CONSORT recommendations.

**Methodology and Principal Findings:**

Ninety peer reviewed journal articles reporting the results of acupuncture trials were selected at random from a wider sample frame of 266 papers. Papers published in three distinct time periods (1994–1995, 1999–2000 and 2004–2005) were compared. Assessment criteria were developed directly from CONSORT and STRICTA checklists. Papers were independently assessed for quality of reporting by two assessors, one of whom was blind to information which could have introduced systematic bias (e.g. date of publication). We detected a statistically significant increase in the reporting of CONSORT items for papers published in each time period measured. We did not, however, find a difference between the number of STRICTA items reported in journal articles published before and 3 to 4 years following the introduction of STRICTA recommendations.

**Conclusions and Significance:**

The results of this study suggest that general standards of reporting for acupuncture trials have significantly improved since the introduction of the CONSORT statement in 1996, but that quality in reporting details specific to acupuncture interventions has yet to change following the more recent introduction of STRICTA recommendations. Wider targeting and revision of the guidelines is recommended.

## Introduction

Adequate reporting of clinical trials improves transparency, and aids interpretation and replication of studies. In an attempt to combat a history of poor reporting the Consolidated Standards of Reporting Trials (CONSORT) were introduced in 1996 [1] and revised five years later [2]. The acupuncture-specific Standards for Reporting Interventions in Controlled Trials of Acupuncture (STRICTA) were compiled and published in late 2001/early 2002 [3]–[5].

The STRICTA guidelines expand on CONSORT Item 4 (i.e. interventions) for use by authors of acupuncture trials. They encourage reporting of intervention details thought to be useful for critical analysis and replication. Five journals have adopted the STRICTA guidelines so far, all of which focus on research within complementary and alternative medicine (CAM); Acupuncture in Medicine, Complementary Therapies in Medicine, Journal of Alternative and Complementary Medicine, Medical Acupuncture, and Clinical Acupuncture and Oriental Medicine (now ceased publication).

Whilst it has previously been shown that the introduction of CONSORT led to improved reporting within adopting journals [6], to date there has been no formal assessment of the impact of the STRICTA guidelines on acupuncture trial reporting.

The present study was therefore designed to assess the impact of the introduction of the STRICTA and CONSORT guidelines on the reporting of acupuncture trials. We wanted to find out how well information pertaining to STRICTA and CONSORT items were reported in the literature and whether reporting had improved over time.

## Methods

### Study design

**Figure 1:**
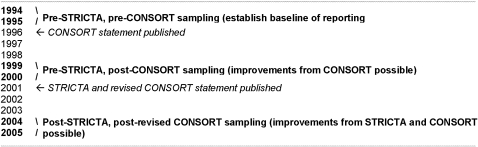
Rationale for time periods of study selection.

We used a before-and-after design to investigate possible changes in quality of reporting between three distinct two-year time periods. Figure 1 illustrates our rationale for choosing date ranges. We aimed to establish a baseline for reporting quality, and track changes over time after publication of CONSORT and STRICTA. We anticipated that a period of 3 to 4 years following the publication of reporting standards would be sufficient for assimilation amongst the academic community. This took account of suggestions that a previous attempt to evaluate the impact of CONSORT just 12–18 months after publication was premature [6].

### Searching and Selection

A systematic and comprehensive literature search was conducted with the aim of identifying published prospective randomised controlled acupuncture trials. No resources were available to search literature published in languages other than English. Trials were identified using MEDLINE, AMED, EMBASE and Cochrane Central Register of Controlled Trials, the search strategy is detailed in Appendix S1. Relevant articles were identified from their title and abstract. Articles were eligible if they reported prospective randomised controlled trials (RCTs) of any design involving acupuncture needle insertion on humans, and were published in an English language journal between three date ranges: 1994–1995; 1999–2000; and 2004–2005. Studies that were published multiple times were included only once, using the most definitive paper for the trial. Abstracts in conference proceedings were excluded. Self-contained short reports such as letters and brief communications were included.

Potentially eligible papers were randomly selected for each of the three time periods using a computer program (SPSS Inc., Chicago Illinois). Articles were then re-assessed for eligibility after the full text version had been obtained. If a paper referred to methods or results that were published elsewhere, attempts were made to obtain the associated papers. If an associated paper was unobtainable or not published in English, the study was discarded.

### Data abstraction

The following general information was abstracted by an unblinded assessor; journal name, publishing house, year of publication, type of journal, the condition under study, type of control, number of participants randomised and article length. For trials with more than two arms, reviewers picked a comparator intervention over a waitlist or no treatment. If there were multiple comparator interventions, minimal acupuncture or sham acupuncture was selected over a non-acupuncture control, and minimal acupuncture over sham acupuncture. Medical journals which had no particular focus on CAM were classified according to whether or not they were general (e.g. *The Lancet*) or covered a specific topic area (e.g. *Pain*). Journals concerned primarily with CAM research were classified by type according to whether or not they had adopted the STRICTA guidelines (applied to 2004–2005 only). Standardised article page length was estimated by dividing the word count of each article by 1300, a typical full page word count of an article published by the *British Medical Journal*.

#### STRICTA assessment checklist

**Figure 2:**
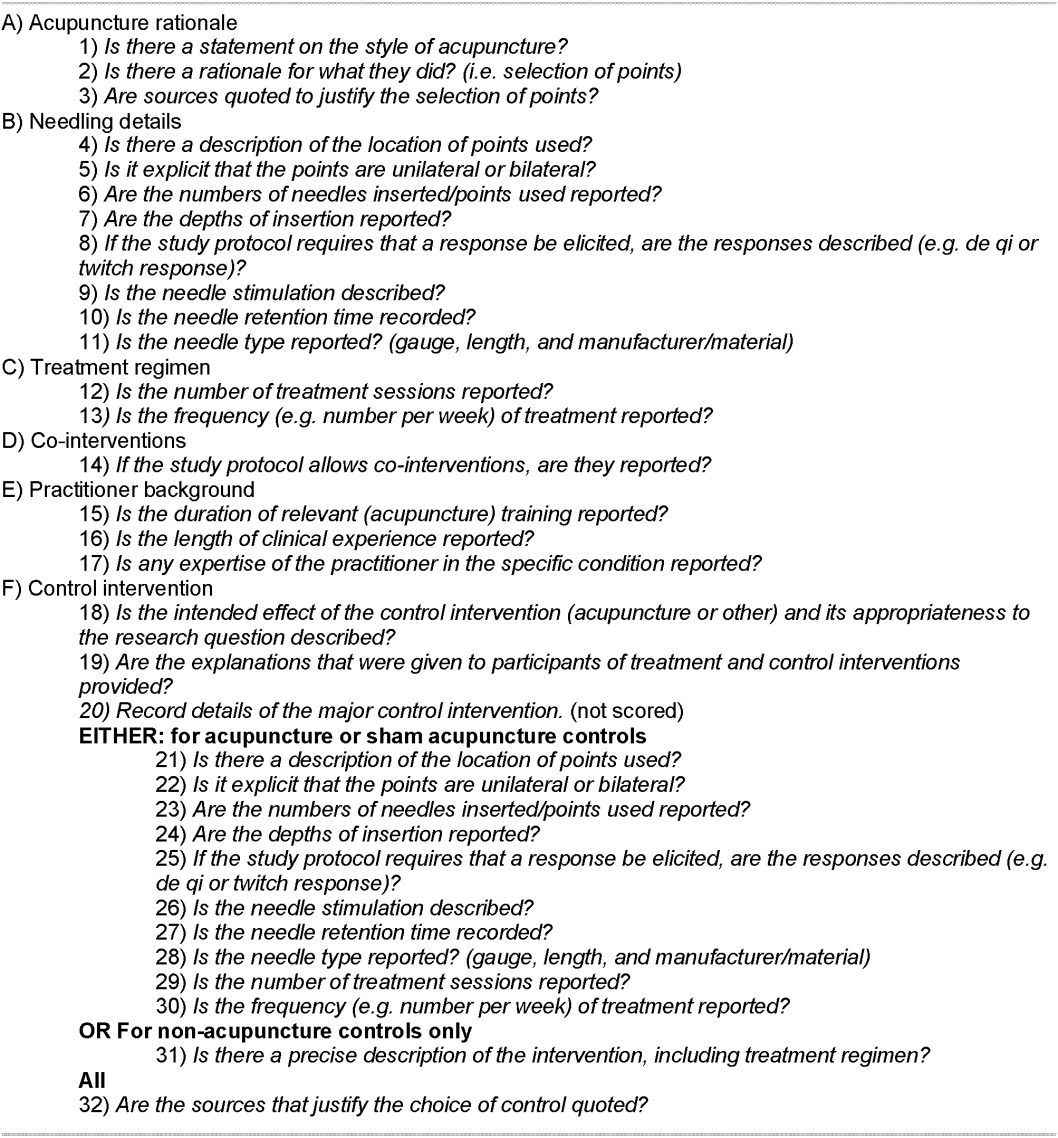
STRICTA checklist used to evaluate studies.

We converted the STRICTA guidelines into a reporting quality assessment checklist involving 30 items for trials incorporating an acupuncture control group, and 21 items for trials in which the comparator did not involve acupuncture. Trials were assessed on items that were relevant to the study design. Items were closely worded to the original recommendations and rephrased as a series of questions, to which the answer could simply be given as ‘yes’ or ‘no’ (Figure 2). To ensure correct interpretation, the two assessors (SLP and SJR) discussed the wording of each item in detail. Where disagreement on the meaning of an item became apparent the wording of the item was revised following consultation with an author of the STRICTA guidelines (HM).

#### CONSORT assessment checklist

**Figure 3:**
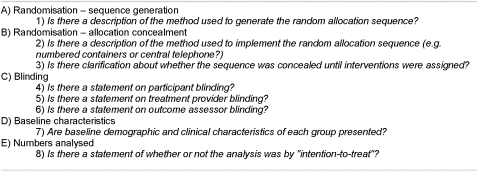
CONSORT checklist used to evaluate studies.

We chose five categories from the CONSORT statement to identify any change in general quality of reporting for acupuncture trials over time. These were among the items selected by previous evaluation studies of the CONSORT statement [7]–[9] because they relate to potential sources of systematic bias [10]–[13] and were present in both the original and revised versions of the checklist. We then developed eight ‘yes’/‘no’ items (Figure 3), worded so that emphasis was placed on quality of reporting rather than adequacy of trial design. To examine the effect of our choice of equal weighting for each of these 8 items we conducted a post-hoc sensitivity analysis. For this we reweighted our chosen CONSORT items equally for the 5 categories by reducing the weight of each of the three blinding items to 1/3 of a point and the two allocation concealment items to ½ a point each (Figure 3) for a total score of 5 rather than a total score of 8.

#### Training of assessors

Two assessors (SLP and SJR), both experienced health service researchers, underwent training on the newly developed assessment checklists. The purpose of the training was to ensure consistency in interpretation and scoring. Initially this involved joint discussion of five research articles not included in the study due to their publication date, for which agreement was reached on the scoring of STRICTA and CONSORT items. Ten papers from the study sample were then randomly selected (stratified by date) and independently scored by both assessors. Following this, inter-rater reliability was calculated and disagreements were resolved by joint discussion with a third assessor (HM). These ten papers were included in the analysis.

#### Blinding

Efforts were made to guard against the possible introduction of systematic bias. In order to assess whether knowledge of publication period, journal type or authorship might affect scoring, all papers given to SJR had this information removed. This was achieved by censoring all pertinent material with a black marker pen or blank paper prior to photocopying. SJR also remained unaware of the three date ranges from which papers were drawn. Blinding of the other assessor (SLP) was not possible due to practical reasons, and she was already familiar with the research literature relating to acupuncture.

### Allocation of papers

All eligible papers that remained were allocated equally between the two assessors by HM using the random sample feature of SPSS. Randomisation was stratified in order to ensure that each assessor received roughly equivalent numbers of papers from each time period. To test concordance following training, the two assessors also received a further 9 identical papers (stratified by date). Each assessor remained unaware which papers had been duplicated for this purpose. Again these were independently scored and later compared in order to estimate inter-rater reliability, bringing the total number of papers jointly assessed to 19. Inconsistencies in scores for these papers were subsequently resolved by HM, who served as an adjudicator.

### Study sample size

As a pilot, 9 papers published outside the study periods (6 before STRICTA publication and 3 after) were randomly selected from a MEDLINE search and scored according to the STRICTA checklist (see Figure 2). A difference of 13.4% (SD 22.5) in items reported was seen between the two time periods. It was estimated that 40 papers per time period would be needed to see this level of difference with 80% power at the 5% significance level (PS Power, Vanderbilt Biostatistics, Nashville). We estimated that 10% of the studies would not meet our eligibility criteria once the full paper was obtained (attrition). This gave us a sample size of 45 papers per time period.

### Statistics

Data were summarised for each time period. The publication details of studies excluded after randomisation were compared with included studies to assess for selection bias using Chi-Square or t-tests. We calculated the proportion of articles reporting each STRICTA item, item subgroup and all items combined before (1994–1995 and 1999–2000) and after (2004–2005) publication of the guidelines, and reported differences as percentage reported with binomial 95% confidence intervals. We also present the percentage, and percentage difference of STRICTA and CONSORT items reported for each of the three time periods with binomial 95% confidence intervals. We repeated these methods on one post-hoc sensitivity analysis testing the effect of re-weighting the CONSORT items.

Concordance between reviewers was assessed using Cohen's kappa statistic for each item and for all items combined. Success of blinding was reported, together with a comparison of assessors in terms of scoring over time, again using percentage reported and with binomial 95% confidence intervals.

Linear regression was used to analyse potential predictors of better reporting. Independent variables were the publication date, page length, type of journal, publishing house and CONSORT score. The dependent variable was the number of STRICTA items reported in each article.

## Results

### Sample selection and flow

**Figure 4:**
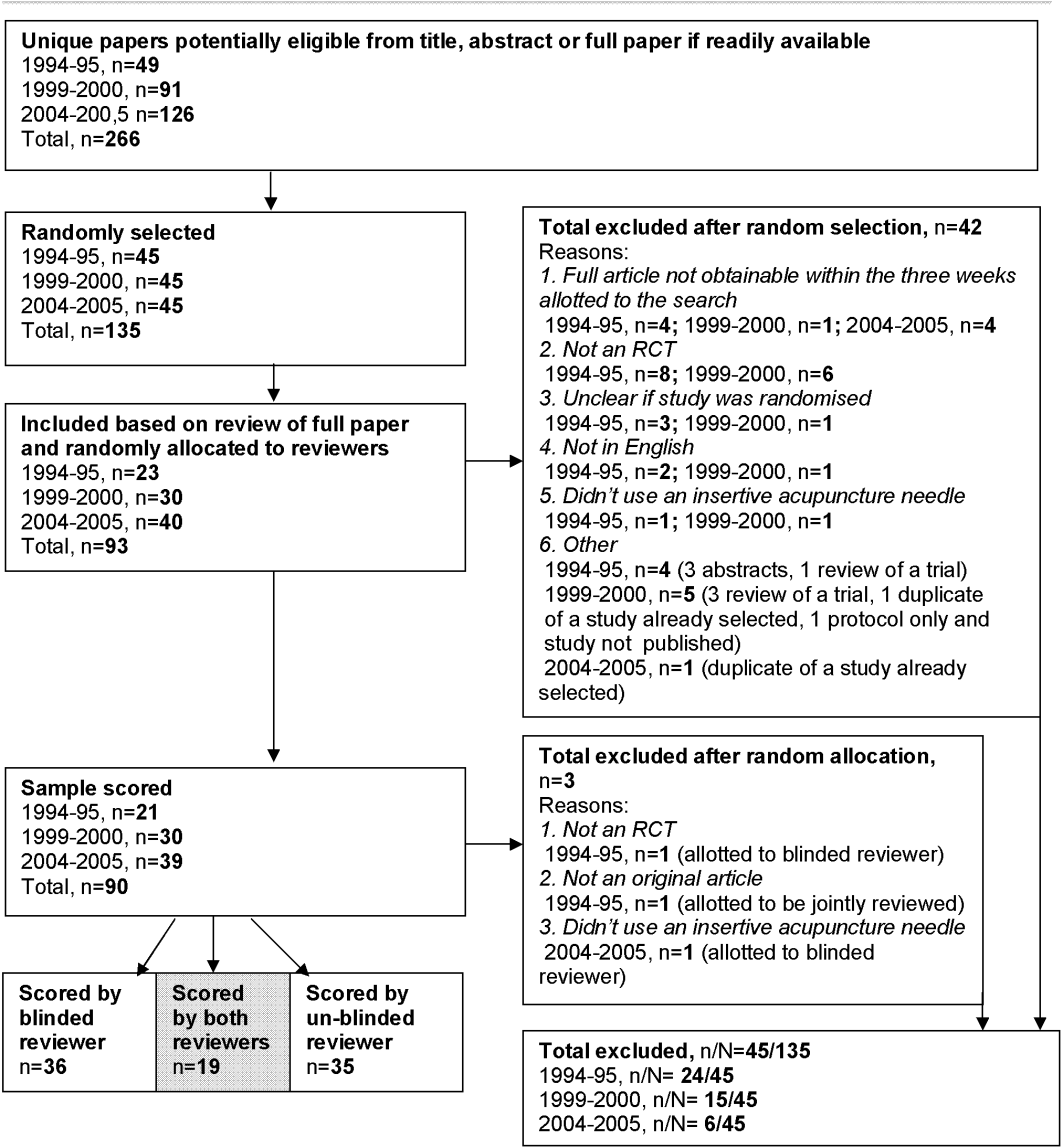
Flowchart.

Two hundred and sixty-six research articles were identified initially as meeting our inclusion criteria (Figure 4). We randomly sampled 135 of these, stratified equally for each of the three time periods, and then attempted to obtain and reassess each full text article. This led to the exclusion of a further 45 papers for various reasons. Most commonly; 19 papers failed to describe a randomized controlled trial, 9 articles could not be obtained, and 9 papers were not complete reports of original research. Articles that were incorrectly classified as RCTs by the search databases tended to come from the two earlier time periods. This resulted in a difference in the proportion of papers excluded between periods. In total 90 eligible research articles were retained for scoring (n = 21 for 1994–1995, n = 30 for 1999–2000, n = 39 for 2004–2005). See Appendix S2 for a bibliography of included papers.

### Study characteristics

The characteristics of all studies from articles sampled at random (those included in the analysis and those excluded) are presented in Table 1. The vast majority of studies were published either in speciality journals or journals which focus on CAM. Only 7% (3/45) of studies published since the introduction of STRICTA were published in STRICTA-adopting journals. Twenty-nine percent of trials investigated the effect of acupuncture on neurological conditions (mostly headache and stroke) with musculoskeletal pain and post-operative pain/recovery the next two most studied areas. Some kind of needling control, either non-penetrating sham needles, sham locations, minimal needles or a combination of the three were the most frequent choice of control. The 90 studies included in the analysis were published in 52 different journals from 30 publishing houses. Seventeen papers were published in 3 Chinese/Taiwanese journals.

**Table 1:** Characteristics of evaluated studies and those excluded following random sampling, n (%) or n (IQ-range)

	1994–1995			1999–2000			2004–2005			Overall		
	Included	Excluded	Total	Included	Excluded	Total	Included	Excluded	Total	Included	Excluded	Total
	*N = 21*	N = 24*	N = 45*	*N = 30*	*N = 15* a	*N = 45* a	*N = 39*	*N = 6* a	*N = 45* a	*N = 90*	*N = 45* a	*N = 135* a
**Journal details**												
Specialty (non-CAM)	8 (38)	14 (58)	22 (49)	16 (53)	4 (27)	20 (44)	24 (62)	4 (67)	28 (62)	48 (53)	22 (49)	70 (52)
CAM (non-STRICTA)	12 (57)	10 (42)	22 (49)	11 (37)	11 (73)	22 (49)	9 (23)	1 (17)	10 (22)	32 (36)	22 (49)	54 (40)
Gen medical (non-CAM)	1 (5)	0 (0)	1 (2)	3 (10)	0 (0)	3 (7)	4 (10)	0 (0)	4 (9)	8 (9)	0 (0)	8 (6)
CAM (STRICTA)	-	-	-	-	-	-	2 (5)	1 (17)	3 (7)	2 (2)	1 (1)	3 (2)
Total	*n = 21*	*n = 24*	*n = 45*	*n = 30*	*n = 15*	*n = 45*	*n = 39*	*n = 6*	*n = 45*	*n = 90*	*n = 45*	*n = 135*
**Conditions studied**												
Neurology	7 (33)	4 (20)	11 (28)	10 (33)	5 (33)	15 (33)	9 (23)	3 (50)	12 (27)	26 (29)	12 (29)	38 (29)
Other	3 (14)	5 (25)	8 (20)	3 (10)	3 (20)	6 (13)	16 (41)	1 (17)	17 (38)	22 (24)	9 (22)	31 (24)
Musculoskeletal pain/rheumatology	4 (19)	4 (20)	8 (20)	4 (13)	4 (27)	8 (18)	7 (18)	0 (0)	7 (16)	15 (17)	8 (20)	23 (18)
Post-op pain & recovery	2 (10)	3 (15)	5 (13)	8 (27)	0 (0)	8 (18)	3 (8)	2 (33)	5 (11)	13 (14)	5 (12)	18 (14)
Drug addiction	4 (19)	3 (15)	7 (18)	2 (7)	2 (13)	4 (9)	1 (3)	0 (0)	1 (2)	7 (8)	5 (12)	12 (9)
Healthy physiology	1 (5)	1 (5)	1 (3)	3 (10)	1 (7)	4 (9)	3 (8)	0 (0)	3 (7)	7 (8)	2 (5)	9 (7)
Total	*n = 21*	*n = 20*	*n = 40*	*n = 30*	*n = 15*	*n = 45*	*n = 39*	*n = 6*	*n = 45*	*n = 90*	*n = 41*	*n = 131*
**Type of control**												
Other acupuncture	9 (43)	5 (31)	14 (38)	12 (40)	5 (50)	17 (43)	13 (33)	0 (0)	13 (32)	34 (38)	10 (36)	44 (37)
Drugs/other	6 (29)	6 (38)	12 (32)	4 (13)	2 (20)	6 (15)	5 (13)	1 (50)	6 (17)	15 (17)	9 (32)	24 (20)
Care as usual/waitlist/no intervention	2 (10)	1 (6)	3 (8)	7 (23)	1 (10)	8 (20)	10 (26)	0 (0)	10 (24)	19 (21)	2 (7)	21 (18)
Non-penetrating sham needles	4 (19)	2 (13)	6 (16)	5 (17)	1 (10)	6 (15)	6 (15)	1 (50)	7 (17)	15 (17)	4 (14)	19 (16)
Physical therapy	0 (0)	2 (13)	2 (5)	2 (7)	1 (10)	3 (8)	5 (13)	0 (0)	5 (12)	7 (8)	3 (11)	10 (8)
Total	*n = 21*	*n = 16*	*n = 37*	*n = 30*	*n = 10*	*n = 40*	*n = 39*	*n = 2*	*n = 41*	*n = 90*	*n = 28*	*n = 118*
**Median no. participants randomized**	41	49	43	51	60	54	72	51	72	57	56	57
	(25–85)	(29–104)	(29–88)	(37–111)	(22–96)	(30–104)	(40–111)	(36–526)	(40–114)	(33–103)	(30–106)	(32–104)
	*n = 21*	*n = 16*	*n = 37*	*n = 30*	*n = 13*	*n = 43*	*n = 39*	*n = 5*	*n = 44*	*n = 90*	*n = 34*	*n = 124*

CAM, Complementary and alternative medicine; IQ, interquartile

Percentages may not total 100 due to rounding

a some data unavailable

As previously noted, 45 studies (i.e. 33%) were excluded after random sampling for failing to meet inclusion criteria (Figure 4), with differential losses between time periods. Because of this we examined study characteristics for evidence of selection bias. Excluded studies in the 1999–2000 time period were more likely to have been published in non-CAM journals than included studies (Pearson's Chi-square 5.3, p = 0.02, 1 df). No other differences were noted.

### Variation in STRICTA reporting over time

Reporting of STRICTA items remained constant over time (Table 2), in 2004–2005 only 53.4% of applicable items were reported (95% CI, 50.2 to 56.6%). There was evidence of a slight improvement in reporting between 1999–2000 and 2004–2005 (difference 5.3%, 95% CI, 0.4 to 10.1%).

**Table 2:** Percentage of STRICTA items reported and between time-period differences

Year	Percentage reported	Differences (95% CI)	
	(n/N) %, 95%CI	1999–2000	2004–2005
1994–1995^a^	(263/513) 51.3, 46.9 to 55.6	−3.2 (−8.8 to 2.4)	+2.1 (−3.3 to 7.5)
1999–2000^a^	(350/727) 48.1, 44.5 to 51.7	-	+5.3 (0.4 to 10.1)
Combined^b^	(613/1240) 49.4, 46.6 to 52.2	-	+4.0 (−0.2 to 8.2)
2004–2005^c^	(502/940) 53.4, 50.2 to 56.6	-	-

a pre-STRICTA

b 1994–1995 and 1999–2000 combined

c post-STRICTA

Note: a positive difference indicates a reporting improvement over time

The differences in the percentage of individual items reported in studies published before and after the implementation of STRICTA are presented in Table 3. The least reported items (reported ≤15% of the time) were Section E (practitioner background) and F19 (explanations of control to patients). The most frequently reported items (reported ≥70% of the time) were A1 (statement on style of acupuncture), A2 (rationale), B4 (description of location of points) and F18 (intended effect of control intervention). There was evidence that two items were reported more frequently after the implementation of STRICTA; items A2 (rationale) and B11 (intervention needle type). No inference could be made for a third item F25 (control response to needle) due to a very wide confidence interval. There were trends of more frequent reporting post-STRICTA in several other items. When items were combined into categories to improve power, before-and-after differences were observed in sections A (acupuncture rationale), and B (needling details). We did not observe a significant difference between the scores when all the items before and after the publication of the STRICTA guidelines were combined (difference 4.0%, 95% CI, −0.2 to 8.2%).

**Table 3:** Reporting of STRICTA items and change in reporting over time

Item	Items from		Items from		Difference (95% CI)^c^
	Pre-STRICTA papers^a^		Post-STRICTA papers^b^		
	n/N	(%)	n/N	(%)	
*A. Acupuncture rationale*					
1. Statement on acu style	33/51	(64.7)	29/39	(74.4)	+9.7 (−9.3 to 28.7)
2. Rationale	32/51	(62.7)	32/39	(82.1)	+19.4 (1.5 to 37.3)
3. Justification of rationale	21/51	(41.2)	21/39	(53.8)	+12.6 (−8.1 to 33.3)
**Total Section A**	**86/153**	**(56.2)**	**82/117**	**(70.0)**	**+13.8 (2.4 to 25.2)**
*B. Needling details (intervention)*					
4. Location of points	44/51	(86.3)	35/39	(89.7)	+3.4 (−10.0 to 16.8)
5. Unilateral/bilateral	27/51	(52.9)	28/39	(71.8)	+18.9 (−0.8 to 38.6)
6. Number of needles	26/51	(51.0)	26/39	(66.7)	+15.7 (−4.5 to 35.9)
7. Depths of insertion	22/51	(43.1)	17/39	(43.6)	+0.5 (−20.2 to 21.2)
8. Response to needle (if applicable)	9/19	(47.4)	17/23	(73.9)	+26.5 (−2.2 to 55.2)
9. Needle stimulation	32/51	(62.7)	21/39	(53.8)	−8.9 (−29.4 to 11.6)
10. Retention time	26/51	(51.0)	17/39	(43.6)	−7.4 (−28.1 to 13.3)
11. Needle type	11/51	(21.6)	17/39	(43.6)	+22.0 (2.8 to 41.2)
**Total Section B**	**197/376**	**(52.4)**	**178/296**	**(60.1)**	**+7.7 (0.2 to 15.2)**
*C. Treatment regimen*					
12. Number of sessions	33/51	(64.7)	20/39	(51.3)	−13.4 (−33.8 to 7.0)
13. Frequency of sessions	37/51	(72.6)	25/39	(64.1)	−8.5 (−27.9 to 10.9)
**Total Section C**	**70/102**	**(68.6)**	**45/78**	**(57.7)**	**−10.9 (−25.1 to 3.3)**
*D. Co-interventions*					
14. Co-intervention (if applicable)	5/8	(62.5)	4/5	(80.0)	
**Total Section D**	**5/8**	**(62.5)**	**4/5**	**(80.0)**	**+17.5 (−31.0 to 66.0)**
*E. Practitioner background*					
15. Duration of training	1/51	(2.0)	4/39	(10.3)	+8.3 (−3.4. to 21.7)
16. Length of clinical experience	3/51	(5.9)		(15.4)	+9.5 (−6.6 to 24.4)
17. Expertise on condition	2/51	(3.9)	4/39	(10.3)	+6.3 (−7.3 to 20.0)
**Total Section E**	**6/153**	**(3.9)**	**14/117**	**(12.0)**	**+8.0 (0.0 to 15.5)**
*F. Control Intervention*					
18. Intended effect of control	37/51	(72.5)	30/39	(76.9)	+4.4 (−13.6 to 22.4)
19. Pt explanations of control	4/51	(7.8)	6/39	(15.4)	+7.6 (−5.9 to 21.1)
*Acupuncture control only*					
21. Location of points	20/30	(66.7)	16/21	(76.2)	+9.5 (−15.3 to 34.3)
22. Unilateral/bilateral	18/30	(60.0)	14/21	(66.7)	+6.7 (−20.0 to 33.4)
23. Number of needles	16/30	(53.3)	11/21	(52.4)	−0.9 (−28.7 to 26.9)
24. Depths of insertion	19/30	(63.3)	13/21	(61.9)	−1.4 (−28.4 to 25.6)
25. Response to needle (if applicable)	2/4	(50.0)	3/3	(100.0)	+50.0 (1.0 to 99.0)
26. Needle stimulation	22/30	(73.3)	14/21	(66.7)	−6.6 (−32.2 to 19.0)
27. Retention time	19/30	(63.3)	9/21	(42.9)	−20.4 (−47.7 to 6.9)
28. Needle type	12/30	(40.0)	10/21	(47.6)	+7.6 (−20.0 to 35.2)
29. Number of sessions	20/30	(66.7)	13/21	(61.9)	−4.8 (−31.6 to 22.0)
30. Frequency of sessions	23/30	(76.7)	14/21	(66.7)	−10.0 (−35.2 to 15.2)
*Non-acupuncture control*					
31. Regimen	10/21	(47.6)	10/18	(55.6)	+8.0 (−23.4 to 39.4)
*All controls*					
32. Sources justifying control	27/51	(52.9)	16/39	(41.0)	−11.9 (−32.5 to 8.7)
**Total Section F**	**249/448**	**(55.6)**	**179/327**	**(54.7)**	**−0.9 (−8.0 to 6.2)**
**TOTAL STRICTA**	**613/1240**	**(49.4)**	**502/940**	**(53.4)**	**+4.0 (−0.2 to 8.2)**

a Pre-STRICTA denotes studies published in 1994–1995 and 1999–2000 combined

b Post-STRICTA denotes those published in 2004–2005

c A positive difference indicates an improvement in post-STRICTA scores

### Variation in CONSORT reporting over time

Reporting of the selected CONSORT items showed evidence of improvement over time (Table 4) with a difference of 10.6% (95% CI, 1.8 to 19.3%) in scores 3–4 years after the original CONSORT statement and a subsequent improvement of 17.2% (95% CI, 9.0 to 25.4%) 3–4 years after publication of the revised version. Fifty-one percent of the selected CONSORT items were reported in 2004–2005 (95% CI, 45.4 to 56.5%). A post-hoc sensitivity analysis reweighting the scoring made no significant difference to these estimates.

**Table 4:** Percentage of CONSORT items reported and between time-period differences

Year	Percentage reported	Differences (95% CI)	
	(n/N) %, 95%CI	1999–2000	2004–2005
1994–1995^a^	(39/168) 23.2, 16.8 to 29.6	+10.6 (1.8 to 19.3)	+27.8 (19.3 to 36.3)
1999–2000^b^	(81/240) 33.8, 27.8 to 39.8	-	+17.2 (9.0 to 25.4)
Combined^c^	120/408) 29.4, 25.0 to 33.8	-	+21.6 (14.5 to 28.7)
2004–2005^d^	(159/312) 51.0, 45.4 to 56.5	-	-

a pre-CONSORT

b post-CONSORT, pre-revised CONSORT

c 1994–1995 and 1999–2000 combined

d post-revised CONSORT

Note: a positive difference indicates a reporting improvement over time

### Inter-rater reliability

There was a high degree of concordance (kappa ≥0.8) [14] between assessors in terms of their scoring for the majority of STRICTA (17/31) and CONSORT (6/8) checklist items. However, there was ‘poor’ to ‘fair’ agreement (kappa <0.4) for the following 6/31 STRICTA items: A1 (statement on the style of acupuncture); B8 (description of needling response); C12 (number of treatment sessions reported); F28 (control needle type); F30 (frequency of control treatments); and F31 (description of non-acupuncture control treatment). Agreement for the remaining items were in the ranges of ‘moderate’ for 4/31 STRICTA items (kappa ≥0.4 to <0.6), and ‘substantial’ for 4/31 STRICTA and 2/8 CONSORT items (kappa ≥0.6 to <0.8) [14].

Overall the assessors showed substantial agreement [14] in their scoring of both checklists in 19 papers, with kappa statistics of 0.78 for STRICTA and 0.84 for CONSORT. There were no differences in the level of inter-rater reliability between the 10 papers used for training and the 9 assessed later, indicating that the reviewers scored consistently throughout the study.

### Influence of blinding

Procedures used to blind one of the assessors (SJR) to key information appeared generally successful. He reported identifying probable dates of publication for just 4 of the 54 papers assessed, which resulted from incomplete masking, and knew which journals had published 2 of the papers because of familiarity with article layout.

Taking the results from each reviewer separately, for the year 1995–1996 we found some evidence of higher STRICTA scoring by the unblinded reviewer (difference 13.4%, 95% CI 3.0 to 23.8%). We found no difference for the other time periods or for the assessment of CONSORT scores.

### Predictors of STRICTA reporting

None of the variables we examined (journal type, publication date, publication house, CONSORT score or page length) were significant predictors of a higher STRICTA score, and they accounted for very little of the variance. Examination of the residuals demonstrated that the model was a good fit.

## Discussion

### Summary of findings

This study is the first systematic investigation on the impact of the STRICTA guidelines on reporting of acupuncture trials. While we found evidence that reporting of two of the 32 items had improved since publication of the guidelines in early 2002, overall we found little meaningful evidence of change over time. None of the variables we looked at in our regression model were significant predictors of improved STRICTA scores. To set a baseline in general reporting of trials our study encompassed a time period that spanned the development of the CONSORT statement. In the same articles we found significant improvements between each time period in the reporting of CONSORT items pertaining to bias. We noted that by 2004–2005 reporting of STRICTA remained unchanged from 10 years previously at 51%. In the 2004–2005 papers we found similar levels of reporting for CONSORT items, although this had improved significantly from previous time periods. The 2004–2005 reporting levels of CONSORT items in this study are lower than another study of non-acupuncture trials [9] and higher than those of pediatric CAM trials [15].

### Strengths and weaknesses

Our study was rigorously conducted and methodologically sound but contains limitations which may have affected our results and interpretation. Like the CONSORT evaluations by Devereaux et al [7] and Moher et al [15] we sampled RCTs in the English-language literature regardless of whether the publishing journal had adopted STRICTA or CONSORT or the journals impact factor. In contrast, other studies of CONSORT have selected a more purposive sample of papers from CONSORT-adopting and non-adopting journals [6], [8]–[9]. To prevent selection bias and increase confidence in our ability to generalise results we randomly selected studies and randomly allocated them to each reviewer. We comprehensively and systematically trained on scoring the checklists and scrutinized concordance between assessors on 19 papers, which we found to be substantial [14] and broadly consistent with those found in other CONSORT evaluations [6]–[8]. To assess whether scoring was influenced by knowledge of the publication date and other factors we blinded one reviewer and assessed the quality of the masking and differences in scoring between the blinded and unblinded assessor. We found some evidence that the unblinded reviewer gave higher scores to her allocation of 1994–1995 papers. This suggests that knowledge of publication date or other factors on the part of the unblinded assessor may have introduced systematic bias for the first time period. However as there was no evidence of scoring differences between reviewers for the other time periods we feel this finding is likely spurious and due to variation in the small number of papers assessed by each reviewer for this time period.

A possible shortcoming is the potential lack of power in our study. We estimated that a sample size of 40 papers for each arm would show a difference in STRICTA scores, but attrition was higher than expected at one-third of papers and highest for the earlier time-points. This may have prevented us seeing a statistically significant difference in some of our end points; indeed, visual inspection of the confidence intervals for between group differences for STRICTA indicates trends towards improvement over time. In argument against a lack of power being a limiting factor we did see a difference in CONSORT scores at each time period however the very poor quality of reporting of CONSORT items in 1994–1995 (23.2%) may well have left greater scope for improvement than the 51.3% of STRICTA items reported at that time period.

The most obvious limitation (and one inherent in all similar studies) is that we turned a checklist into an unvalidated scoring scale [16], a purpose for which it was not designed. Although we trained on and pre-tested our scales, we do not know the extent of item discrimination, which lends an additional unknown quantity of error to our results. Although concordance between the assessors was high overall and found not to have biased the between-group differences, it was evident that even after substantial training we were unable to agree on the meaning of a small number of STRICTA items which could have affected the results.

Some studies examining the effect of CONSORT have evaluated all [6], [15] or most [17]–[18] of the checklist items, while others have selected only a few items [7]–[9]. In this study we also evaluated a sub-set of CONSORT items rather than the entire checklist. We chose to do this because the publication dates of our assessed articles spanned two versions of CONSORT and evaluation of either one or the other list would have introduced a systematic bias by disadvantaging articles following a different version of the guideline. We selected the 8 items in 5 categories (Figure 3) on the basis that they had been included in other studies evaluating a subset of CONSORT items [7]–[9] and reflected important methodological considerations that have been shown bias to outcomes [10]–[13]. They also had to be items that were very similar in both versions of the CONSORT checklist. There is a possibility that this selectivity resulted in us missing the ‘true’ proportion of CONSORT items reported, though it could be argued that greater importance should be placed on factors shown to affect results.

In our analysis we assumed equal weight for all scored items for both the STRICTA and CONSORT checklists. To test whether our weighting choice had any effect on the data we conducted a post-hoc sensitivity analysis reweighting our chosen CONSORT items equally for each of the 5 sections of items, rather than each item. We found no evidence of any differences between estimates of scores at each time period or between-group differences scored on either method and conclude that the weighting did not bias our results.

Due to resource limitations we did not search for or evaluate papers in languages other than English; but we did include papers written in English that were published in foreign journals and 20% of our included papers were from China and Taiwan. However, given the quantity of acupuncture research available in countries such as China we may have missed a substantial body of foreign literature from which to draw our sample. Authors have analysed the reporting of positive results in acupuncture [19] and low accessibility of studies [20] from foreign countries but there are few specific data available on the relative quality and quality of reporting of acupuncture trials in non-English language journals. This is an area for worthwhile future study. It remains unknown whether searching in foreign language journals would have altered the constitution of our sample or results.

### Meaning of our results

We found an improvement in the number of CONSORT items reported between each period of CONSORT publication up to 8–9 years after initial publication, but did not see similar improvements in the STRICTA scores before and after the publication of STRICTA. The STRICTA guidelines were published only 3–4 years before our study endpoint and these results may indicate that we conducted the STRICTA evaluation too soon, in that insufficient time has elapsed in order for the guidelines to have any obvious effect. A similar concern was raised by the authors of another before-and-after study of CONSORT reporting conducted 12–18 months after the release of the first guideline [6].

It is reasonable to assume that more article space would encourage higher levels of reporting and Mills et al [9] found the number of pages weakly correlated with improved reporting of CONSORT. However in our model we did not find standardised page length predictive of increased reporting of STRICTA items. Neither was the CONSORT score associated with an improved STRICTA score, a factor we conclude is due to the lack of awareness of the STRICTA guidelines. Overall quality of reporting has substantially increased since the introduction of CONSORT, but reporting of the acupuncture intervention specific guidelines appears independent and remains unaffected.

An important finding was that only two of the 39 papers assessed after the publication of STRICTA were published in a STRICTA-adopting journal and the majority of acupuncture clinical trials were published in a wide variety of non-CAM specialist journals. Among these journals we believe that CONSORT has been more widely promoted than STRICTA, resulting in general improvements in reporting quality, but not for acupuncture-specific items. Further exploration into CONSORT adherence and STRICTA awareness by journals publishing acupuncture trials is warrented. A study conducted by the present authors found that some acupuncture trialists report that STRICTA items originally included in submitted manuscripts are removed during the editorial process [21]. This suggests that STRICTA guidelines may need to be promoted more widely amongst journals which are not specific to CAM.

Our lack of concordance between reviewers for some items despite extensive discussion implies that part of the STRICTA checklist is ambiguous. Authors may also fail to appreciate the underlying need for reporting some items.

### Conclusions

Our results suggest that more time may need to elapse in order to observe potential improvements from the STRICTA guidelines. This study should therefore be repeated in the future reviewing a greater number of studies to counteract any possible limitations regarding lack of power. Consideration should also be given to validating the checklists for use as scoring instruments. We have discovered that very few acupuncture trials are published in journals that have formally adopted STRICTA and further strategies for promotion should be considered with these findings in mind. Previous evaluations of CONSORT reporting [22] have contributed to the review process of the guidelines [2] and we think our findings demonstrate there is also a good case for rethinking and clarifying some items contained in STRICTA.

## Supporting Information

Appendix S1 (0.05 MB DOC)

Appendix S2 (0.05 MB DOC)
