# Supplementary material for: A Systematic Evaluation of the Impact of STRICTA and CONSORT Recommendations on Quality of Reporting for Acupuncture Trials
Source: PLoS One. 2008 Feb 13;3(2):e1577. doi: 10.1371/journal.pone.0001577 (PMC2216683; doi:10.1371/journal.pone.0001577)
Supplement: Appendix S1 — (0.05 MB DOC) [file pone.0001577.s001.doc]

**Appendix 1. Search strategy**

**MEDLINE**

1 ACUPUNCTURE/

2 exp Acupuncture Therapy/

3 ELECTROACUPUNCTURE/

4 acupunctur$.ti,ab.

5 electroacupunctur$.ti,ab.

6 electro-acupunctur$.ti,ab.

7 auriculoacupunctur$.ti,ab.

8 percutaneous electrical nerve stimulation.ti,ab.

9 PENS.ti,ab.

10 acupoint$.ti,ab.

11 meridian$.ti,ab.

12 dry needling.ti,ab.

13 or/1-12

14 RANDOMIZED CONTROLLED TRIAL.pt.

15 CONTROLLED CLINICAL TRIAL.pt.

16 RANDOMIZED CONTROLLED TRIALS.sh.

17 RANDOM ALLOCATION.sh.

18 DOUBLE BLIND METHOD.sh.

19 SINGLE BLIND METHOD.sh.

20 or/14-19

21 (ANIMALS not HUMAN).sh.

22 20 not 21

23 CLINICAL TRIAL.pt.

24 exp CLINICAL TRIALS/

25 (clin$ adj25 trial$).ti,ab.

26 ((singl$ or doubl$ or trebl$ or tripl$) adj25 (blind$ or mask$)).ti,ab.

27 PLACEBOS.sh.

28 placebo$.ti,ab.

29 random$.ti,ab.

30 RESEARCH DESIGN.sh.

31 or/23-30

32 31 not 21

33 32 not 22

34 22 or 33

35 13 and 34

Limit by: English, dates

**EMBASE**

1 exp ACUPUNCTURE/

2 acupunctur$.ti,ab.

3 electroacupunctur$.ti,ab.

4 electro-acupunctur$.ti,ab.

5 auriculoacupunctur$.ti,ab.

6 percutaneous electrical nerve stimulation.ti,ab.

7 PENS.ti,ab.

8 acupoint$.ti,ab.

9 meridian$.ti,ab.

10 dry needling.ti,ab.

11 or/1-10

12 clinical trial/

13 randomized controlled trial/

14 randomization/

15 single blind procedure/

16 double blind procedure/

17 crossover procedure/

18 placebo/

19 randomi?ed controlled trial$.tw.

20 rct.tw.

21 random allocation.tw.

22 randomly allocated.tw.

23 allocated randomly.tw.

24 (allocated adj2 random).tw.

25 single blind$.tw.

26 double blind$.tw.

27 ((treble or triple) adj blind$).tw.

28 placebo$.tw.

29 prospective study/

30 or/12-29

31 case study/

32 case report.tw.

33 abstract report/ or letter/

34 or/31-33

35 30 not 34

36 11 and 35

Limit by: Human, English, dates

**AMED**

1 exp Acupuncture/

Limit by; English and dates.

**COCHRANE CENTRAL REGISTER OF CONTROLLED TRIALS**

Acupuncture (title, abstract or keyword)

OR

dry needling (title, abstract or keyword)

OR

electroacupunctur* (title, abstract or keyword)

OR

electro-acupunctur* (title, abstract or keyword)

OR

auriculo-acupunctur* (title, abstract or keyword)
