# Supplementary material for: A Systematic Evaluation of the Impact of STRICTA and CONSORT Recommendations on Quality of Reporting for Acupuncture Trials
Source: PLoS One. 2008 Feb 13;3(2):e1577. doi: 10.1371/journal.pone.0001577 (PMC2216683; doi:10.1371/journal.pone.0001577)
Supplement: Appendix S2 — (0.05 MB DOC) [file pone.0001577.s002.doc]

**Appendix 2. Bibliography of papers reviewed**

**1994-1995**

1.     Arnetz BB, Berg M, Anderzen I, Lundeberg T, Haker E. (1995) A nonconventional approach to the treatment of "environmental illness". J Occup Environ Med 37(7): 838-844.

2.     Avants SK, Margolin A, Chang P, Kosten TR, Birch S. (1995) Acupuncture for the treatment of cocaine addiction. investigation of a needle puncture control. J Subst Abuse Treat 12(3): 195-205.

3.     Chen D, Gong D, Zhai Y. (1994) Clinical and experimental studies in treating diabetes mellitus by acupuncture. J Tradit Chin Med 14(3): 163-166.

4.     Ernst E, Resch KL, Fialka V, RitterDittrich D, Alcamioglu Y, et al. (1995) Traditional acupuncture for reflex sympathetic dystrophy: A randomised, sham-controlled, double-blind trial. Acupuncture in Medicine 13(2): 78-80.

5.     Hesse J, Mogelvang B, Simonsen H. (1994) Acupuncture versus metoprolol in migraine prophylaxis: A randomized trial of trigger point inactivation.[see comment]. J Intern Med 235(5): 451-456.

6.     Kelleher CJ, Filshie J, Burton G, Khullar V, Cardozo LD. (1994) Acupuncture and the treatment of irritative bladder symptoms. Acupuncture in Medicine 12(1): 9-12.

7.     Kho KH. (1995) The impact of acupuncture on pain in patients with reflex sympathetic dystrophy. The Pain Clinic 8(1): 59-61.

8.     Konefal J, Duncan R, Clemence C. (1994) The impact of the addition of an acupuncture treatment program to an existing metro-dade county outpatient substance abuse treatment facility. Journal of Addictive Diseases 13(3): 71-99.

9.     Lao L, Bergman S, Langenberg P, Wong RH, Berman B. (1995) Efficacy of chinese acupuncture on postoperative oral surgery pain. Oral Surg 79(4): 423-428.

10.     Lao L, Bergman S, Anderson R, Langenberg P, Wong RH, et al. (1994) The effect of acupuncture on post-operative oral surgery pain: A pilot study. Acupuncture in Medicine 12(1): 13-17.

11.     Li Q, Cao S, Xie G, Gan Y, Ma H, et al. (1994) Combined traditional chinese medicine and western medicine. relieving effects of chinese herbs, ear-acupuncture and epidural morphing on postoperative pain in liver cancer. Chin Med J 107(4): 289-294.

12.     Lipton DS, Brewington V, Smith M. (1994) Acupuncture for crack-cocaine detoxification: Experimental evaluation of efficacy. J Subst Abuse Treat 11(3): 205-215.

13.     McIndoe AK, Young K, Bone ME. (1995) A comparison of acupuncture with intra-articular steroid injection as analgesia for osteoarthritis of the hip. Acupuncture in Medicine 13(2): 67-70.

14.     Molsberger A, Hille E. (1994) The analgesic effect of acupuncture in chronic tennis elbow pain. Br J Rheumatol 33(12): 1162-1165.

15.     Pang H. (1994) 52 cases of apoplexy treated with scalp acupuncture by the slow-rapid reinforcing-reducing method. J Tradit Chin Med 14(3): 185-188.

16.     Shafshak TS. (1995) Electroacupuncture and exercise in body weight reduction and their application in rehabilitating patients with knee osteoarthritis. Am J Chin Med 23(1): 15-25.

17.     Takeda W, Wessel J. (1994) Acupuncture for the treatment of pain of osteoarthritic knees. Arthritis Care Res 7(3): 118-122.

18.     Tian L, Yuan S, Ba E, Chen H, Zhou Z. (1995) Composite acupuncture treatment of mental retardation in children. J Tradit Chin Med 15(1): 34-37.

19.     Wang WK, Hsu TL, Chang HC, Wang YY. (1995) Effect of acupuncture at tsu san li (st-36) on the pulse spectrum. Am J Chin Med 23(2): 121-130.

20.     Xinsheng L, Hairui S. (1994) Clinical comparison of the acupuncture treatment of cerebral palsy with standard and 'special points' of the scalp. Am J Acupunct 22(3): 215-219.

21.     Yang X, Liu X, Luo H, Jia Y. (1994) Clinical observation on needling extrachannel points in treating mental depression. J Tradit Chin Med 14(1): 14-18.

**1999-2000**

1.     Ahmed HE, White PF, Craig WF, Hamza MA, Ghoname ES, et al. (2000) Use of percutaneous electrical nerve stimulation (PENS) in the short-term management of headache. Headache 40(4): 311-315.

2.     Avants SK, Margolin A, Holford TR, Kosten TR. (2000) A randomized controlled trial of auricular acupuncture for cocaine dependence.[see comment]. Arch Intern Med 160(15): 2305-2312.

3.     Barlas P, Robinson J, Allen J, Baxter GD. (2000) Lack of effect of acupuncture upon signs and symptoms of delayed onset muscle soreness. Clin Physiol 20(6): 449-456.

4.     Bullock ML, Kiresuk TJ, Pheley AM, Culliton PD, Lenz SK. (1999) Auricular acupuncture in the treatment of cocaine abuse. A study of efficacy and dosing. J Subst Abuse Treat 16(1): 31-38.

5.     Chen CH, Chen TW, Weng MC, Wang WT, Wang YL, et al. (2000) The effect of electroacupuncture on shoulder subluxation for stroke patients. Kaohsiung J Med Sci 16(10): 525-532.

6.     Coe TR. (1999) The effect of acupuncture on pain and swelling after day case molar teeth extraction under general anaesthesia. Ambul Surg 7(1): 45-49.

7.     David J, Townsend S, Sathanathan R, Kriss S, Dore CJ. (1999) The effect of acupuncture on patients with rheumatoid arthritis: A randomized, placebo-controlled cross-over study.[see comment]. Rheumatology (Oxford) 38(9): 864-869.

8.     Gao S, Zhao D, Xie Y. (1999) A comparative study on the treatment of migraine headache with combined distant and local acupuncture points versus conventional drug therapy. Am J Acupunct 27(1-2): 27-30.

9.     Gupta S, Francis JD, Tillu AB, Sattirajah AI, Sizer J. (1999) The effect of pre-emptive acupuncture treatment on analgesic requirements after day-case knee arthroscopy.[see comment]. Anaesthesia 54(12): 1204-1207.

10.     Haker E, Egekvist H, Bjerring P. (2000) Effect of sensory stimulation (acupuncture) on sympathetic and parasympathetic activities in healthy subjects. J Auton Nerv Syst 79(1): 52-59.

11.     Heikkila H, Johansson M, Wenngren BI. (2000) Effects of acupuncture, cervical manipulation and NSAID therapy on dizziness and impaired head repositioning of suspected cervical origin: A pilot study. Manual Ther 5(3): 151-157.

12.     Karst M, Rollnik JD, Fink M, Reinhard M, Piepenbrock S. (2000) Pressure pain threshold and needle acupuncture in chronic tension-type headache--a double-blind placebo-controlled study. Pain 88(2): 199-203.

13.     Kitade T, Ohyabu H. (2000) Analgesic effects of acupuncture on pain after mandibular wisdom tooth extraction. Acupunct Electrother Res 25(2): 109-115.

14.     Kloster R, Larsson PG, Lossius R, Nakken KO, Dahl R, et al. (1999) The effect of acupuncture in chronic intractable epilepsy. Seizure 8(3): 170-174.

15.     Lao L, Bergman S, Hamilton GR, Langenberg P, Berman B. (1999) Evaluation of acupuncture for pain control after oral surgery: A placebo-controlled trial. Arch Otolaryngol Head Neck Surg 125(5): 567-572.

16.     Linde, Carlsson JY, Dahlof CG. (2000) Impact of acupuncture as add-on therapy to pharmacological treatment of migraine: A pilot study. Pain Clinic 12(3): 247-252.

17.     Liu L. (2000) Acupuncture treatment of bulbar palsy--a report of 54 cases. J Tradit Chin Med 20(1): 30-32.

18.     Long WJ. (2000) Acupuncture treatment of acute lumbar sprain: A controlled study in 238 patients. International Journal of Clinical Acupuncture 11(1): 61-64.

19.     Lu DP, Lu GP, Reed JF,3rd. (2000) Acupuncture/acupressure to treat gagging dental patients: A clinical study of anti-gagging effects. Gen Dent 48(4): 446-452.

20.     Lun X, Rong L. (2000) Twenty-five cases of intractable cutaneous pruritus treated by auricular acupuncture. J Tradit Chin Med 20(4): 287-288.

21.     Shen J, Wenger N, Glaspy J, Hays RD, Albert PS, et al. (2000) Electroacupuncture for control of myeloablative chemotherapy-induced emesis: A randomized controlled trial.[see comment]. JAMA 284(21): 2755-2761.

22.     Stener-Victorin E, Waldenstrom U, Nilsson L, Wikland M, Janson PO. (1999) A prospective randomized study of electro-acupuncture versus alfentanil as anaesthesia during oocyte aspiration in in-vitro fertilization. Hum Reprod 14(10): 2480-2484.

23.     Tao Y. (2000) Eighty cases of injury of the superior cluneal nerve treated by electroacupuncture. J Tradit Chin Med 20(2): 132-133.

34.     Wan Q. (2000) Auricular-plaster therapy plus acupuncture at zusanli for postoperative recovery of intestinal function. J Tradit Chin Med 20(2): 134-135.

25.     Wang RR, Tronnier V. (2000) Effect of acupuncture on pain management in patients before and after lumbar disc protrusion surgery--a randomized control study. Am J Chin Med 28(1): 25-33.

26.     Wedenberg K, Moen B, Norling A. (2000) A prospective randomized study comparing acupuncture with physiotherapy for low-back and pelvic pain in pregnancy. Acta Obstet Gynecol Scand 79(5): 331-335.

27.     White A, Ernst E. (1999) The effect of auricular acupuncture on the pulse rate: An exploratory randomised controlled trial. Acupuncture in Medicine 17(2): 86-88.

28.     White AR, Resch KL, Chan JC, Norris CD, Modi SK, et al. (2000) Acupuncture for episodic tension-type headache: A multicentre randomized controlled trial. Cephalalgia 20(7): 632-637.

29.     Yang C, Yan H. (1999) Observation of the efficacy of acupuncture and moxibustion in 62 cases of chronic colitis. J Tradit Chin Med 19(2): 111-114.

30.     Zang J. (1999) 80 cases of peripheral facial paralysis treated by acupuncture with vibrating shallow insertion. J Tradit Chin Med 19(1): 44-47.

**2004-2005**

1.     Che-Yi C, Wen CY, Min-Tsung K, Chiu-Ching H. (2005) Acupuncture in haemodialysis patients at the quchi (LI11) acupoint for refractory uraemic pruritus. Nephrol Dial Transplant 20(9): 1912-1915.

2.     Cristian A, Katz M, Cutrone E, Walker RH. (2005) Evaluation of acupuncture in the treatment of parkinson's disease: A double-blind pilot study. Mov Disord 20(9): 1185-1188.

3.     DiLorenzo L, Traballesi M, Morelli D, Pompa A, Brunelli S, et al. (2004) Hemiparetic shoulder pain syndrome treated with deep dry needling during early rehabilitation: A prospective, open-label, randomized investigation. J Musculoskeletal Pain 12(2): 25-34.

4.     Elden H, Ladfors L, Olsen MF, Ostgaard HC, Hagberg H. (2005) Effects of acupuncture and stabilising exercises as adjunct to standard treatment in pregnant women with pelvic girdle pain: Randomised single blind controlled trial.[see comment]. BMJ 330(7494): 761.

5.     Emmons SL, Otto L. (2005) Acupuncture for overactive bladder: A randomized controlled trial. Obstet Gynecol 106(1): 138-143.

6.     Forbes A, Jackson S, Walter C, Quraishi S, Jacyna M, et al. (2005) Acupuncture for irritable bowel syndrome: A blinded placebo-controlled trial. World J Gastroenterol 11(26): 4040-4044.

7.     Fu B, Lun X, Gong Y. (2005) Effects of the combined therapy of acupuncture with herbal drugs on male immune infertility - A clinical report of 50 cases. Journal of Traditional Chinese Medicine 25(3): 186-189.

8.     Gronlund MA, Stenevi U, Lundeberg T. (2004) Acupuncture treatment in patients with keratoconjunctivitis sicca: A pilot study. Acta Ophthalmol Scand 82(3 Pt 1): 283-290. .

9.     Habek D, Barbir A, Habek JC, Janculiak D, BobicVukovic M. (2004) Success of acupuncture and acupressure of the pc 6 acupoint in the treatment of hyperemesis gravidarum. Forsch Komplementarmed Klass Naturheilkd 11(1): 20-23.

10.     Hsu CH, Hwang KC, Chao CL, Chang HH, Chou P. (2005) Electroacupuncture in obese women: A randomized, controlled pilot study. J Womens Health (Larchmt) 14(5): 434-440.

11.     Hsu CH, Hwang KC, Chao CL, Lin JG, Kao ST, et al. (2005) Effects of electroacupuncture in reducing weight and waist circumference in obese women: A randomized crossover trial. Int J Obes (Lond) 29(11): 1379-1384.

12.     Huang ST, Chen GY, Lo HM, Lin JG, Lee YS, et al. (2005) Increase in the vagal modulation by acupuncture at neiguan point in the healthy subjects. Am J Chin Med 33(1): 157-164.

13.     Humaidan P, Stener-Victorin E. (2004) Pain relief during oocyte retrieval with a new short duration electro-acupuncture technique--an alternative to conventional analgesic methods.[see comment]. Hum Reprod 19(6): 1367-1372.

14.     Joos S, Brinkhaus B, Maluche C, Maupai N, Kohnen R, et al. (2004) Acupuncture and moxibustion in the treatment of active crohn's disease: A randomized controlled study. Digestion 69(3): 131-139.

15.     Kawakita K, Shichidou T, Inoue E, Nabeta T, Kitakouji H, et al. (2004) Preventive and curative effects of acupuncture on the common cold: A multicentre randomized controlled trial in Japan. Complement Ther Med 12(4): 181-188.

16.     Kim YS, Lee SH, Jung WS, Park SU, Moon SK, et al. (2004) Intradermal acupuncture on shen-men and nei-kuan acupoints in patients with insomnia after stroke. Am J Chin Med 32(5): 771-778.

17.     Kou W, Bell JD, Gareus I, Pacheco-Lopez G, Goebel MU, et al. (2005) Repeated acupuncture treatment affects leukocyte circulation in healthy young male subjects: A randomized single-blind two-period crossover study. Brain Behav Immun 19(4): 318-324.

18.     Kvist LJ, Wilde Larsson B, Hall-Lord ML, Rydhstroem H. (2004) Effects of acupuncture and care interventions on the outcome of inflammatory symptoms of the breast in lactating women. Int Nurs Rev 51(1): 56-64.

19.     Kvorning N, Holmberg C, Grennert L, Aberg A, Akeson J. (2004) Acupuncture relieves pelvic and low-back pain in late pregnancy. Acta Obstet Gynecol Scand 83(3): 246-250.

20.     Lin Q, Li X, Han J, Leng J. (2005) Electro-acupuncture treatment for the upper segment ureterolithiasis under B-ultrasonography. J Tradit Chin Med 25(1): 13-15.

21.     Liu Y. (2004) Treatment of pseudobulbar paralysis by scalp acupuncture and sublingual needling. J Tradit Chin Med 24(1): 26-27.

22.     Meissner W, Weiss T, Trippe RH, Hecht H, Krapp C, et al. (2004) Acupuncture decreases somatosensory evoked potential amplitudes to noxious stimuli in anesthetized volunteers. Anesth Analg 98(1): 141-147.

23.     Melchart D, Hager S, Hager U, Liao J, Weidenhammer W, et al. (2004) Treatment of patients with chronic headaches in a hospital for traditional chinese medicine in germany. A randomised, waiting list controlled trial. Complement Ther Med 12(2-3): 71-78.

24.     Melchart D, Streng A, Hoppe A, Brinkhaus B, Witt C, et al. (2005) Acupuncture in patients with tension-type headache: Randomised controlled trial. BMJ 331(7513): 376-382.

25.     Muller R, Giles LGF. (2005) Long-term follow-up of a randomized clinical trial assessing the efficacy of medication, acupuncture, and spinal manipulation for chronic mechanical spinal pain syndromes. Journal of Manipulative & Physiological Therapeutics 28(1): 3-11.

26.     Neri I, Allais G, Schiapparelli P, Blasi I, Benedetto C, et al. (2005) Acupuncture versus pharmacological approach to reduce hyperemesis gravidarum discomfort. Minerva Ginecol 57(4): 471-475.

27.     Pariente J, White P, Frackowiak RS, Lewith G. (2005) Expectancy and belief modulate the neuronal substrates of pain treated by acupuncture. Neuroimage 25(4): 1161-1167.

28.     Park J, White AR, James MA, Hemsley AG, Johnson P, et al. (2005) Acupuncture for subacute stroke rehabilitation: A sham-controlled, subject- and assessor-blind, randomized trial. Arch Intern Med 165(17): 2026-2031.

29.     Seki T, Iwasaki K, Arai H, Sasaki H, Hayashi H, et al. (2005) Acupuncture for dysphagia in poststroke patients: A videofluoroscopic study. J Am Geriatr Soc 53(6): 1083-1084.

30.     Streitberger K, Diefenbacher M, Bauer A, Conradi R, Bardenheuer H, et al. (2004) Acupuncture compared to placebo-acupuncture for postoperative nausea and vomiting prophylaxis: A randomised placebo-controlled patient and observer blind trial.[see comment]. Anaesthesia 59(2): 142-149.

31.     Usichenko TI, Dinse M, Hermsen M, Witstruck T, Pavlovic D, et al. (2005) Auricular acupuncture for pain relief after total hip arthroplasty - a randomized controlled study. Pain 114(3): 320-327.

32.     Vickers AJ, Feinstein MB, Deng GE, Cassileth BR. (2005) Acupuncture for dyspnea in advanced cancer: A randomized, placebo-controlled pilot trial [ISRCTN89462491]. BMC Palliative Care Aug 18(4): 5.

33.     Wang L. (2004) Clinical observation on acupuncture treatment in 35 cases of diabetic gastroparesis. J Tradit Chin Med 24(3): 163-165.

34.     Wayne PM, Krebs DE, Macklin EA, Schnyer R, Kaptchuk TJ, et al. (2005) Acupuncture for upper-extremity rehabilitation in chronic stroke: A randomized sham-controlled study. Arch Phys Med Rehabil 86(12): 2248-2255.

35.     Witt C, Brinkhaus B, Jena S, Linde K, Streng A, et al. (2005) Acupuncture in patients with osteoarthritis of the knee: A randomised trial.[see comment]. Lancet 366(9480): 136-143.

36.     Xue CC, Dong L, Polus B, English RA, Zheng Z, et al. (2004) Electroacupuncture for tension-type headache on distal acupoints only: A randomized, controlled, crossover trial. Headache 44(4): 333-341.

37.     Yokoyama M, Sun X, Oku S, Taga N, Sato K, et al. (2004) Comparison of percutaneous electrical nerve stimulation with transcutaneous electrical nerve stimulation for long-term pain relief in patients with chronic low back pain. Anesth Analg 98(6): 1552-1556.

38.     Yue Z, Zhenhui Y. (2005) Ulcerative colitis treated by acupuncture at jiaji points (EX-B2) and tapping with plum-blossom needle at sanjiaoshu (BL22) and dachangshu (BL 25)--a report of 43 cases. Journal of Traditional Chinese Medicine 25(2): 83-84.

39.     Zeng X, Lei L, Lu Y, Wang Z. (2005) Treatment of heroinism with acupuncture at points of the du channel. Journal of Traditional Chinese Medicine 25(3): 166-170.
